# Epidemiology of methicillin resistant *Staphylococcus pseudintermedius* in guide dogs in Finland

**DOI:** 10.1186/s13028-015-0129-8

**Published:** 2015-07-17

**Authors:** Thomas Grönthal, Matti Ollilainen, Marjut Eklund, Heli Piiparinen, Veera Gindonis, Jouni Junnila, Leena Saijonmaa-Koulumies, Riitta Liimatainen, Merja Rantala

**Affiliations:** Department of Equine and Small Animal Medicine, Faculty of Veterinary Medicine, University of Helsinki, P.O. Box 57, 00014 Helsinki, Finland; 4Pharma Ltd., Ahventie 4, 02170 Espoo, Finland; Guide Dog School of the Finnish Federation of the Visually Impaired, Siltaniitynkuja 1, 01260 Vantaa, Finland

**Keywords:** *Staphylococcus*, MRSA, MRSP, Epidemiology, Prevalence, Risk factor

## Abstract

**Background:**

Methicillin resistant *Staphylococcus pseudintermedius* (MRSP) and *Staphylococcus aureus* (MRSA) are common multi-drug resistant (MDR) bacteria in dogs. In 2012–2013 three dogs of the Guide Dog School of the Finnish Federation of the Visually Impaired were found to be MRSP positive. Guide dogs have regular contact with each other during their first year of life and prolonged contact when in training. Since dogs are placed in different parts of Finland after training, there is a risk for national spread of MDR bacteria. In this study the prevalence of MRSP and MRSA, as well as the risk factors for MRSP were determined in the Finnish guide dog population. MRSP isolates were investigated using molecular methods and compared to the earlier isolates.

**Results:**

Out of 132 tested dogs 4 were MRSP positive thus giving the prevalence estimate of 3% (95% CI: 1–8%) for MRSP in the target population. MRSA was not detected (prevalence estimate 0%, 95% CI: 0–3%). Risk factors associated with MRSP were being a breeding bitch (OR = 8.4; 95% CI: 1.1–64.1, *P* = 0.012), the number of veterinary visits (OR = 1.23; 95% CI: 1.0–1.5, *P* = 0.025) and number of antimicrobial courses (OR = 1.63; 95% CI: 1.0–2.55; *P* = 0.035). Identified MRSP isolates belonged to five different sequence types (ST45, 71, 402, 403 and 404). All ST71 isolates carried SCC*mec* II-III, while the SCC*mec* type of the ST45 and ST402 (a single locus variant of ST45) isolates were non-typeable with the method used.

**Conclusions:**

MRSP and MRSA had low prevalence in the studied dog population despite the close contact between dogs, and the MRSP population was heterogenic. Antimicrobial therapy and veterinary visits are risk factors for MRSP even among a small case group.

**Electronic supplementary material:**

The online version of this article (doi:10.1186/s13028-015-0129-8) contains supplementary material, which is available to authorized users.

## Background

*Staphylococcus pseudintermedius* belongs to the normal microbiota of dogs and is an opportunistic pathogen [1]. The bacterium can cause a plethora of infections in dogs and is also capable of causing infections in humans [2]. Since the mid 2000’s methicillin resistant *S. pseudintermedius* (MRSP) isolates have become more common [3, 4]. This has raised concern of emerging antimicrobial resistance and infections that are difficult to cure with available antimicrobials [2]. MRSP has spread clonally in Europe and North America [5]. The predominant European clone, ST71(MLST)-t02(*spa*)-II-III(SCC*mec*), has been determined to be the principal MRSP clone in Sweden [6] and has also been observed in Finland, where it caused a large outbreak in a veterinary teaching hospital [7]. Of other staphylococci, *Staphylococcus aureus* is a common human pathogen, but is less prevalent in dogs [8]. Nevertheless, methicillin resistant *S. aureus* (MRSA), traditionally a nosocomial pathogen of humans, is also found in dogs and can cause infections in them [9]. MRSA may also be more readily transmitted between dogs and owners than MRSP [10, 11].

The Guide Dog School of the Finnish Federation of the Visually Impaired provides guide dogs to the blind and visually impaired in Finland. In 2012–2013, MRSP was found in routine clinical specimens of three guide dogs, all suffering from atopic dermatitis. The dogs of the Guide Dog School constitute a special group of animals; they are in close contact during training at the Guide Dog School kennel, after which they are sent all over the country. During their service years, they also regularly visit the school’s premises. Thus nationwide risk for spread of resistant bacteria is a valid concern. Due to these factors it was decided that the epidemiology of MRSP and MRSA needed to be determined in this population. The present study was conducted (1) to establish the prevalence of MRSP and MRSA in the Finnish guide dog population, (2) to genotype and compare isolates from the Guide Dog School’s dog population and (3) to detect risk factors for carriage of MRSP.

## Methods

This study consisted of four parts; (1) a cross-sectional prevalence study of MRSP/A in the guide dog population as well as a case–control study to determine the risk factors for MRSP/A, (2) the screening of contact dogs of MRSP positive dogs from the prevalence study, along with extended screening of breeding bitches of the Guide Dog School and (3) the comparison of the MRSP isolates from these dogs. The fourth part of the study included mapping of possible transmission routes as well as longitudinal follow-up of the MRSP-status of four dogs.

### The prevalence and risk factor study

#### The guide dog population

The Guide Dog School (GDS) of the Finnish Federation of the Visually Impaired is situated in the greater Helsinki area in Finland [12]. The school is a nonprofit organization whose mission is to enhance the independence of people who are blind or visually impaired through the use of specially trained dogs. The school breeds the majority of their dogs themselves. Breeding bitches are housed in volunteer families. The bitches give birth at the school’s facilities in a separate ward reserved for this purpose. Puppies spend their first 6 weeks with their dam at the school after which they are weaned and housed in volunteer families. During the first year of their life the dogs have regular (short) appointments at the school. At the age of 13–18 months guide dog candidates are transferred back to the school where they undergo suitability testing. If selected, the dog begins a training period of 20 weeks after which it is placed for service as a guide dog. During their service the dogs spend short periods at the school in training; the GDS’s facilities also serve as a kennel to guide dogs during vacations of their host/hostess. At the time the study was planned, the size of the guide dog population was approximately 330 dogs. Out of these roughly 110 dogs were in training, 200 worked as guide dogs and 20 animals were breeding bitches.

#### Sample size calculation

Sample size for the entire 330 dog population was calculated using EpiTools [13]. The estimated sample size was 152 dogs with an approximated MRSP prevalence of 3% with a ±2% desired precision on a 95% confidence level. The occurrence of MRSP varies significantly between studies depending on used methods, population and geographical origin. Figures between zero and 42 percent have been reported [14–21]. The guide dogs were considered a low risk population, thus the three percent estimate was based on studies that were assessed to represent our target population. These showed a prevalence between 1.5 and 4.5 percent [15–17, 20, 21]. The calculation of sample size for MRSA separately was not considered necessary, since the prevalence of MRSA was expected to be less than that of MRSP.

#### Specimens and data collection

Specimens for the prevalence study were collected between February and November 2014 when the dogs visited the GDS. This method was selected out of convenience instead of random sampling. Three to four sites of each dog were swabbed by using sterile cotton tips (M40 Transsystem Amies Agar Gel without charcoal, Copan Diagnostics Inc., Italy): the first swab was taken from the mucous membranes of the anterior nares and mouth (combined swab), the second swab from the perineal area, and the third from skin abrasions/wounds if present. The specimens were then transferred to the Clinical Microbiology Laboratory at the Faculty of Veterinary Medicine of the University of Helsinki. In the laboratory the specimens were kept refrigerated at 4°C if they arrived outside office hours. All specimens were processed within 2 days of sampling.

To determine the representativeness of the sample population to the target population, the age, sex, breed and dog group variables as well as the exact number of dogs in the whole population and different subgroups were collected from the entire target population. To identify possible risk factors for MRSP, the following data were collected from the dogs of the sample population: breed, sex, age, whether the animal was bred by the GDS or purchased, whether it was a trainee, at service or a breeding bitch, and medical history. Medical history included information on previous skin disease, antimicrobial and other medical treatments within a 12 month period prior to entering the study.

#### Data analysis

The prevalence estimates for MRSP and MRSA were calculated by number of positive specimens divided by total number of collected specimens and presented as percentages. The 95% confidence intervals (95% CI) for the prevalence estimates were determined using an EpiTools calculator [22]. Confidence intervals were reported based on the Wilson score interval due to the low prevalence [23]. The representativeness of the sample population was assessed by using the Fisher’s exact test for sex, breed and dog group, and using an independent two-sample *t* test for age.

Descriptive statistics of the study variables were calculated by MRSP status. To identify risk-factors, the associations of the studied variables (see “Specimen and data collection”) with a positive MRSP result were evaluated with univariable logistic regression models. Due to very few positive MRSP results in the data, the rareness of the events was taken into account in the modelling by applying Firth’s bias adjustment method [24], which maximizes a penalized likelihood function, instead of the standard maximum likelihood function. Odds ratios (OR) with 95% profile likelihood [25] confidence intervals (CI) were calculated to quantify the results. *P* values ≤0.05 were considered statistically significant. All statistical analyses were done using SAS® System for Windows, version 9.3 (SAS Institute Inc., Cary, NC, USA).

### Specimens from contact dogs and breeding bitches

Three contact dogs of MRSP positive guide dogs were screened for MRSP. Because two breeding bitches were observed to be MRSP-positive in the prevalence study, MRSP screening was extended to the rest of dogs of that subpopulation (n = 5). The same technique as for the prevalence study was used when taking these specimens.

### Mapping of transmission routes, and follow-up specimens

Information on possible transmission routes was obtained by questioning the caregiver of the dogs as well as from event logs of the guide dog school. The MRSP statuses of four dogs were followed for over 6 months. Specimens were collected as described above. MRSP decolonization therapy using chlorhexidine washes and/or topical application of fusidic acid to mucous membranes was attempted in three of these dogs.

### Bacteriological analyses

#### Culture, identification and susceptibility testing

Specimens from the same dog were pooled into an enrichment broth (Tryptic Soy Broth supplemented with 6.5% NaCl, Tammer-Tutkan Maljat, Finland) and incubated for 16–22 h at +35.0°C. Then an aliquot of the broth was plated onto a selective agar plate (MRSA Select, BioRad, USA). Plates were incubated up to 48 h at +35.0°C and were inspected daily. Species identification was done by conventional means; as described by Grönthal et al. [7]

Antimicrobial susceptibility was tested by the CLSI disk diffusion method to the following antimicrobials: oxacillin, erythromycin, clindamycin, tetracycline, sulfamethoxazole-trimethoprim, fusidic acid, enrofloxacin, gentamicin and amikacin [26, 27]. SIR categories were assigned based on the breakpoints of the CLSI standard, except for fusidic acid, for which breakpoints of ≥24 mm (susceptible) and ≤18 mm (resistant) were used [28]. *Staphylococcus aureus* ATCC 25923 was used as a quality control strain for susceptibility testing.

#### Molecular typing of the MRSP isolates

Molecular typing was performed for MRSP isolates detected in the prevalence study, MRSP isolates of the contact dogs and for previous MRSP isolates from the guide dogs.

To detect clonal clusters, *mec*A-positive isolates were typed using pulsed-field gel electrophoresis (PFGE) [29] with modifications [7]. All isolates were analyzed with both *SmaI* and *AscI* restriction enzymes. DNA fragments were separated on 1% agarose by using a Chef DR III system (Bio-Rad, USA). The total run time was 22 h; the first-block switch time was 0.1–15 s for 15 h, and the second-block switch time was 15–60 s for 7 h. The voltage for the run was 6 V/cm with an included angle of 120°. PFGE patterns were analyzed using Gel Compar II software (version 6.6, Applied Maths, Belgium). UPGMA based cluster analysis with the Dice similarity coefficient was used with optimization and position tolerance both set at 1%. Isolates were clustered using an 85% similarity cut-off.

DNA for *mec*A-PCR, SCC*mec* and MLST-typing was extracted using a commercial kit (InstaGene Matrix, Bio-Rad, USA) as previously described [30]. Oxacillin resistant *S. pseudintermedius* isolates were confirmed to carry the *mec*A-gene using PCR-primers (*mec*A P4 and *mec*A P7) described by Stegger et al. [31]. Both primers had a concentration of 0.25 µM in a final reaction volume of 20 µl. The PCR was performed with a BioRad CFX96 Real-Time PCR detection system using SsoAdvanced Universal SYBR Green Supermix (Bio-Rad, USA) with initial denaturation at 98.0°C for 2 min, followed by 40 cycles of denaturation (98.0°C for 5 s) and annealing/elongation (60.0°C for 45 s). Finally a denaturation step (98.0°C for 5 s) preceded the melt-curve analysis (65.0°C to 95.0°C in 0.5°C increments) that was used to verify the product. The product had a melting point of 77.5–78.0°C. *Staphylococcus aureus* ATCC 43300 was used as a positive control for *mec*A-testing.

The SCC*mec* cassettes were typed using a previously described multiplex PCR-method [32] with the following modifications: In M-PCR-1 primers γR and γF had a final concentration of 0.3 µM, while all other primers in M-PCR-1 and M-PCR-2 had a final concentration of 0.2 µM. The PCRs were performed with Phire Green HotStart II DNA polymerase (Thermo Scientific, USA) with 200 µM of each dNTP in a reaction. The conditions for the PCRs were as follows: Initial denaturation at 98.0°C for 1 min, followed by 40 cycles of denaturation (98.0°C for 15 s), annealing (56.0°C for 10 s) and elongation (72.0°C for 45 s), and a final elongation for 2 min at 72.0°C. Bands were visualized using SYBR Safe DNA stain (Life Technologies, USA) after electrophoresis in a 1% agarose.

MLST typing was performed according to the method described by Solyman et al. [33] with modifications: Phusion Flash High-Fidelity PCR Master Mix (Thermo Scientific, USA) was used for the reaction; the *tuf*-primers had a final concentration of 0.375 µM each, while all other primers had a concentration of 0.25 µM. The PCR-protocol consisted of a 15 s initial denaturation at 98°C, 30 cycles of denaturation (98°C for 2 s), annealing (52°C for 10 s) and elongation (72°C for 15 s), with final extension for 1 min at 72°C. Each PCR-product was purified using Exonuclease I (Thermo Scientific, USA) and FastAP thermosensitive alkaline phosphatase (Thermo Scientific, USA) according to the manufacturer’s instructions. The sequencing for MLST was performed at a commercial laboratory (Macrogen Inc., Netherlands) with an ABI 3730 XL automated sequencer. The sequences were analyzed using the CLC Main Workbench (version 6.9.1, CLC bio, Denmark) with CLC MLST module (version 1.4.7, CLC bio, Denmark) comparing sequences of the housekeeping genes to the *S. pseudintermedius* MLST database [34].

## Results

### Prevalence and risk factor study

Specimens were taken from 132 dogs of which four were MRSP-positive (prevalence estimate for the population 3%, 95% CI: 1–8%). None of the screened dogs were positive for MRSA (prevalence estimate 0%, 95% CI: 0–3%). The breed and sex of the sample population was representative to the target population, while dog groups and age differed to that of the target population (Table 1).

**Table 1:** Comparison of demographics in the target and sample populations of the Finnish Guide Dog School’s dogs in this study

	Sample population		Target population		Statistical difference
	n = 132	%*	n = 308	%*	*P*-value (method)
Sex					0.92 (Fisher’s Exact test)
Male	65	49	158	51	
Female	67	51	150	49	
Breed					0.84 (Fisher’s Exact test)
Labrador retriever	124	94	287	93	
Other	8	6	21	7	
Dog group					0.01 (Fisher’s Exact test)
Guide dog	56	42	187	61	
Dog in training	68	52	103	33	
Breeding dog	8	6	18	6	

Age	Mean (min–max)	Median	Mean (min–max)	Median	0.03 (t test)
	3.8 (0.2–13.9)	2.1	4.6 (0.2–13.9)	3.9	

* Percent values are rounded.

Skin or ear disease history was reported in 49% of the dogs (range 43–55%; lowest in trainees, highest in guide dogs) and antimicrobial treatment in 52% of the dogs (range 43–60%; lowest in guide dogs, highest in trainees). Logistic regression analyses revealed three risk factors; (1) being a breeding bitch, (2) the number of antimicrobial courses received and (3) the number of veterinary visits. Results from logistic regression are presented in Table 2.

**Table 2:** Risk factors associated with MRSP in the Finnish Guide Dog School population in 2014

	MRSP-pos (*n* = 4)		MRSP-neg (*n* = 128)		Univariable logistic regression	
Categorical variables	*n*	%	*n*	%	Unadjusted OR (95% CI)	Likelihood *P*
Demographics						
Gender: F vs.M	3	75.0	62	48.4	2.48 (0.40–26.13)	0.338
Breed (labrador retriever vs. other)	4	100.0	120	93.8	0.64 (0.06–86.75)	0.779
Dog bought vs. bred by school	0	0.0	13	10.2	0.95 (0.01–9.70)	0.973
Dog group						
Breeding vs. training	2	50.0	6	4.7	8.39 (1.12–64.13)	0.012
Training vs. working	0	0.0	68	53.1	0.16 (0.00–2.01)	
Epidemiological data						
History of ear or skin disease	1	25.0	63	52.1	0.40 (0.04–2.48)	0.328
Antimicrobial treatment*	3	75.0	66	52.8	2.09 (0.33–21.99)	0.441

Continuous variables	*n*	Mean	*n*	Mean	Unadjusted OR (95% CI)	Likelihood *P*
Demographics						
Age	4	5.8	128	3.8	1.18 (0.92–1.49)	0.185
Epidemiological data						
Number of veterinary visits*	4	9.8	128	4.9	1.23 (1.030–1.48)	0.025
Number of antimicrobial courses*	4	2.8	126	1.0	1.63 (1.04–2.55)	0.035

* For the past 12 months. *CI* confidence interval using Firth’s bias adjustment (see text) and *OR* odds ratio.

### Screening of contact dogs and breeding bitches

**Figure 1:**
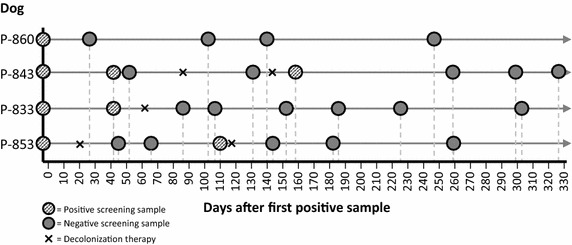
Results from follow-up samples of four dogs in the Finnish Guide Dog School MRSP study. Decolonization therapy using chlorhexidine and/or fusidic acid lasted for 10 days.

Three contact dogs were screened of which two were MRSP positive. These both (dogs P-853 and 2014-887) were living together with the guide dog P-833. The contact P-853 gave a negative result in the first screening, but was observed to be MRSP positive in the second screening a month later (Figure 1). Extended screening of the rest of the breeding bitches (n = 5) revealed no cases of MRSP.

### Comparison of MRSP isolates

**Figure 2:**
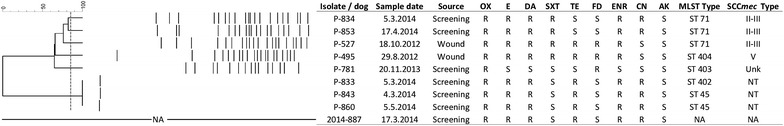
*Sma*I dendogram and antibiograms of the MRSP-isolates investigated in the Finnish Guide Dog School MRSP study. Dogs P-833, P-853 and 2014-887 lived in the same household. *OX* oxacillin, *E* erythromycin, *DA* clindamycin, *SXT* sulphamethoxazole/trimethoprim, *TE* tetracycline, *FD* fucidic acid, *ENR* enrofloxacin, *CN* gentamicin, *AK* amikacin, *R* resistant, *S* susceptible, *NT* non-typeable and *NA* not analyzed. *Unk* the result from the SCC*mec* analysis of isolate P-781 was inconclusive (see text and Additional file 1). The *grey dashed line* indicates the 85% cut-off value.

Characteristics of the nine MRSP isolates are compared in Figure 2: four were from the prevalence study (P-833, P-834, P-843 and P-860), two (P-853 and 2014-887) were from contact dogs of the guide dog P-833 and three were isolates that had been preserved in 2012–2013 from other guide dogs (P-495, P-527 and P-781). All but one isolate (P-781) were multiresistant (resistant to ≥3 antimicrobial groups). The MRSP isolate of a contact dog 2014-887 shared an identical antibiogram with the MRSP of the guide dog P-833, but the other contact’s isolate (P-853) differed from these (Figure 2).

All, except the isolate 2014-887, were available for molecular typing. Of the four isolates from the prevalence study, three (P-833, P-843 and P-860) were non-typeable by *Sma*I restriction but gave identical *Asc*I-restriction profiles (Additional file 2). Both *Sma*I and *Asc*I restriction patterns indicated that the other five isolates were not closely related (Figure 2; Additional File 2).

The four MRSP-isolates from the prevalence study represented three different sequence types (Figure 2). Two were of ST45, while ST71 and ST402 had one representative each. The ST402 strain was a single locus variant of ST45: it had an A_166_ to G substitution in the *pur*A 2 allele sequence compared to ST45. The new *pur*A allele number was 40 [34]. The isolate from dog P-781 was assigned a new sequence type (ST403) being closest to ST41, with a different *cpn60* allele type (13 instead of 25). The isolate from dog P-495 was closest to ST150, but had a different *fdh* allele type (4 instead of 1) and a G_6_ to A substitution in the *sar* 1 allele (assigned *sar* allele number 17). This isolate was designated sequence type ST404 [34]. Isolates belonging to ST71 harbored SCC*mec* II-III. The ST45 and ST402 isolates were non-typeable by the SCC*mec* method used, as only the *mec*A-gene was amplified in M-PCR-1. Isolate P-781 that belonged to ST403 gave an unusual result in the SCC*mec* analysis as it seemed products specific to gene allele’s *ccrA2* and *ccrA4* were amplified in M-PCR-1 (Additional file 1).

### Investigation of transmission routes

Transmission route investigation revealed that dogs P-843 and P-860 had been in direct contact at the GDS. Also, dogs 2014-887 and P-853 lived in the same family as dog P-833. A member of this family worked at a small animal practice, where the dogs had visited. No other apparent temporal or spatial connections between the dogs were found.

### Follow-up specimens

Samples were collected from four MRSP positive dogs. MRSP decolonization therapy was performed on three dogs of which two were guide dogs (P-833 and P-844) and one was a contact of the guide dog P-833. The other contact of dog P-833 was euthanized due to severe illness. The dogs were followed for over 6 months, with four to seven samples taken roughly 2 weeks to 3 months apart. The timing and results of the follow-up samples are presented in Figure 1.

## Discussion

The prevalence of MRSP was determined to be 3% in the Finnish Guide dog population, matching the predicted prevalence. No MRSA positive dogs were discovered, but the true prevalence lies somewhere between 0 and 3%. There are some study limitations to consider. As seen in Table 1, dogs in training were overrepresented at the expense of guide dogs. This discrepancy is explainable by ease of access, as dogs in training were more readily available for sampling at the GDS. This is also a reason to the difference in age, since dogs in training are younger than other dogs. The sample population represented the target population well as for breed and sex, and the targeted sample size (152) was close to the obtained (132). The fact that the sample population was slightly skewed could have impacted the results of the study. Also, as previously reported [7], the screening method used in this study has a limit of detection of ~10 colony forming units for MRSP with a oxacillin minimum inhibitory concentration (MIC) ≥4 µg/ml. Therefore, using this method, MRSPs with very low oxacillin MIC may be missed, and thus the prevalence may be slightly underestimated. As this was a known problem, pure cultures were readily taken from selective plates upon the slightest indication of a suspected colony. In our experience this has yielded many carriers with MRSP-isolates having low oxacillin-MIC (2 µg/ml). In this study a large portion (49%) of the sampled dogs did have a history of skin or ear disease which is a well-known risk factor for MRSP [7, 35]. This potentially increased the likelihood for the presence of MRSP/A carriers in the population and subsequently compensated possible underestimation of the prevalence caused by our screening method. In all, the discrepancies in representativeness and methodology were in our opinion fairly minor and did not significantly impact the result of the study.

Published studies for the prevalence of MRSP or MRSA in Finnish dogs are not available. However, to give some contrast; there were 581 MRSP/A screening specimens investigated in our laboratory in 2014. MRSP was found in 56 of these (9.6%) while MRSA was only found in 5 specimens (0.9%). It is important to note that these specimens were taken from dogs with identifiable risk factors or patients that had been exposed to MRSP or MRSA. Only a few studies exist that have measured the prevalence of MRSP among specific animal groups. In a study of shelter animals by Gingrich et al. [15] in Colorado, USA, the prevalence of MRSP in dogs was 3%, while the prevalence of MRSA in dogs was 0.5%. These numbers coincide quite well with our study. In Slovenia, the prevalence of MRSP (then MRSI) was 1.5% (3/200) in clinically healthy dogs in a community, while MRSA was not discovered [20]. Regarding veterinary hospitals, Hanselman et al. [21] reported a MRSP prevalence of 2.1% and MRSA prevalence of 0.5% in dogs upon admission in Ontario, Canada. A similar study conducted in Hannover, Germany reported a higher MRSP prevalence (7.4%) [36]. Further, Boost et al. [8] investigated the prevalence of *S. aureus* in dogs and their owners in Hong Kong. They found an MRSA prevalence of 0.7% among the dogs investigated, which falls into the 0-3% prevalence estimate in our study and corresponds well to our laboratory data. Further study is however needed to ascertain the prevalence of MRSP and MRSA in the average dog population as well as subpopulations, to identify target groups for preventive measures.

In this study, a dog had a higher risk for MRSP if it was a breeding bitch or had a greater number of antibiotic courses or veterinary visits, but due to the low number of MRSP positive cases our results are only supportive at best. The fact that being a breeding bitch was significant in the risk factor analysis is likely a coincidence due to the low number of dogs in this group. As Figure 1 indicates, one of the MRSP positive breeding bitches (P-860) was only a transient carrier and had likely caught it through contact to another MRSP positive dog (P-843). Extended screening of the breeding bitches did not reveal any new MRSP cases, indicating that it is not a problem among these dogs. It would however be preferable if breeding bitches would remain MRSP negative to avoid the transfer of these bacteria to new generations of guide dogs. Further, there are apparent dependencies between some risk factors: for example, dogs with multiple antimicrobial courses have visited a veterinarian more often. Controlling of these types of confounders with multivariable modelling was not possible in our study due to very small number of MRSP positive dogs. Thus far, there is however compelling evidence to suggest that antimicrobial therapy is a major risk factor for acquiring MRSP/A [7, 36, 37]. Other, previously identified, risk factors for MRSP include hospitalization [35, 36] and repeated veterinary visits [35, 36]. Glucocorticoid therapy has also been implicated as a risk factor for MRSP by some studies [35, 36].

A considerable number (52%) of dogs in this study had received antimicrobial therapy in the twelve months prior to the study. Such a large portion of animals receiving antimicrobials is concerning. Much of the use is likely explained by the large proportion (49%) of dogs having history of skin or ear disease of some sort. Labrador retrievers, which constitute the vast majority of the guide dog population, are known to be predisposed to atopy [38], which exposes them to skin and ear infections and subsequent use of antimicrobials. National, species specific antimicrobial consumption data are not readily available for Finland. In Sweden however, such data has been published in the SVARM report [39]. The amount of oral antimicrobials prescribed per dog corresponded to 563 packages per 1000 dogs in 2009 in Sweden. While many dogs likely received multiple packages that year, the number is still indicative of a high rate of antimicrobial use, comparable to our data. [39]. The use of antimicrobials in dogs could be reduced by prioritizing a healthy skin in breeding choices, which is already part of the GDS’s breeding program.

The MRSP isolates of this study were rather heterogeneous. For example; the isolates belonging to the same clonal lineage, ST71, had different PFGE and antibiogram profiles. This suggests different sources of contagion, possibly outside of the guide dog population. This was somewhat surprising, considering the potential for clonal spread in a kennel environment. It is possible that the MRSP population in Finland is becoming more heterogeneous, perhaps due to a decrease in ST71 occurrence, as has been seen in Sweden [39]. Only two of the dogs (P-843 and P-860) were reported to have been in contact with each other. They both carried ST45 with identical antibiogram and *AscI* PFGE-pulsotype. ST71 and ST45 have previously been found in Finland [7], but three new sequence types (STs 402, 403 and 404) were discovered in this study (Figure 2). The ST403 had a very surprising antibiogram, being resistant only to oxacillin and fusidic acid. In our experience such MRSP isolates are very rare. Osland et al. [40] described MRSP isolates with a similar antibiogram that belonged to ST127. This clone differs in four out of seven MLST loci from our ST403 [34] suggesting a lack of any clonal relationship. Also, the variability of the antibiograms in the ST71 isolates was novel, as one of the three isolates displayed resistance to all antimicrobials but aminoglycosides, while others were susceptible to tetracycline and fusidic acid and amikacin, but resistant to gentamicin. Antibiograms of the ST71 isolates of this study also differed from the ST71 strain causing our veterinary teaching hospital outbreak [7]. The outbreak strain was susceptible only to amikacin and fucidic acid. Based on our unpublished observations, this study, and a previous one by Grönthal et al. [7], ST45 seems to be a noteworthy MRSP-clone in Finland. However, ST45 is not commonly reported in neighboring countries, such as Sweden [6] or Norway [40], nor has it been purported as a major clone on a more global scale [5]. It has however been reported as a major clone in Thailand and Israel [41].

MRSP isolates that are not typeable by *Sma*I PFGE and SCC*mec*, have been described in the Netherlands [42], Thailand and Israel [41], and Australia [43]. The isolates that were non-typeable by SCC*mec* in the study by Laarhoven et al. [42] belonged to MRSP STs 29, 111, 131 and 143, while only ST29 was non-typeable by *SmaI* PFGE. The allelic profiles of these differ from that of ST45. The ST45 and other STs belonging to the clonal complex CC45 harbor a pseudo SCC*mec* element (ΨSCC*mec*_57395_) described by Perreten et al. [41]. The ST45 and ST402 MRSPs isolated in this study will be investigated further in the future to confirm the presence or absence of the pseudo SCC*mec* element.

Based on the results form PFGE, MLST and SCC*mec* analyses, as well as transmission route investigation it seems clear that dogs P-843 and P-860 carried the same MRSP-type. Dog P-833, whose MRSP isolate belonged to ST402 had two contact dogs; P-853 and 2014-887. While the MRSP isolate for dog 2014-887 was not available for further study, it did display an identical antibiogram with isolates belonging to ST45/402 indicating that the isolate also belongs to one of these sequence types. MRSP isolates from the same household are usually similar [10] but some diversity among household pets does occur [42]. The latter was also seen in our results as dog P-853 had a different sequence type; ST71. It is possible that these dogs have acquired MRSP from a veterinary clinic where a family member worked. These dogs did not have apparent risk factors for MRSP carriage.

The results of the follow-up samples varied. Dog P-860, who had been exposed to the long term carrier P-843, was likely a transient carrier or perhaps even it was a case of contamination, as no positive samples were detected during follow-up. Windahl et al. [44] investigated the length of MRSP carriage and the factors that impacted this. Their results indicated that the median length of MRSP carriage is 11 months after clinically apparent infection, but that antimicrobial therapy with a drug to which the bacterium was resistant prolonged carriage time. The study used a cut off value of two consecutive MRSP-negative specimens to determine the dog was MRSP free. However, a longitudinal study of MRSP in households found that the results from MRSP screening varied in the individual dog [42]. This was also the case in our study, as recurrent findings of MRSP in dogs that previously had two MRSP negative specimens (P-843 and P-853) were observed. It is noteworthy however that decolonization therapy was attempted in these cases. This may have altered the dynamics of the dogs’ microbiome causing unpredictable results. Saijonmaa-Koulumies et al. [45] studied the use of fusidic acid as a means to reduce the number of *S. pseudintermedius* (then *S. intermedius*) on mucous membranes of dogs. They found that while therapy did reduce the number of bacteria, the effect was only temporary. It would seem logical to think that MRSP behaved in the same fashion. *S. pseudintermedius* can however be a bacterium difficult to eradicate from dogs, as it is part of the normal microbiota and is carried by a majority of dogs [1]. The use of decolonization therapy for MRSP is a subject of further study.

Based on our findings it seems that neither MRSA nor MRSP are a major concern in the GDS. The standard hygiene practices likely contribute to the low prevalence of MRSA and MRSP at the GDS’s kennels as they are likely higher than in most animal shelters. Also, the GDS’s kennels do not suffer from many problems one could see in rescue kennels, such as overcrowding or severely ill animals [46]. It is however still advised that the GDS should routinely screen dogs with identifiable risk factors, such as skin or ear problems, or dogs that receive a lot of antimicrobials.

## Conclusions

MRSP and MRSA have low prevalence and are not major problems among guide dogs in Finland. The MRSP population in these dogs is epidemiologically heterogeneous and there is little evidence of clonal spread. The number of antimicrobial courses and veterinary visits are identifiable risk factors for MRSP even in a low prevalence canine population.

## Additional files

Additional file 1: Gel image of the products of isolate P-781 and positive controls after SCC*mec* PCR. Description of data: ctr = control; SCC*mec* I ctr: *Staphylococcus aureus* NCTC 10442; SCC*mec* II ctr: *Staphylococcus aureus* ATCC BAA-1720; SCC*mec* III ctr: *Staphylococcus aureus* ATCC BAA-43; SCC*mec* V ctr: *Staphylococcus aureus* JCSC 6944; SCC*mec* VI ctr: *Staphylococcus aureus* ATCC BAA-42. Additional file 2:
*Asc*I PFGE dendogram of the MRSP-isolates investigated. Description of data: All isolates were also investigated using *Sma*I restriction (see text).

## Abbreviations

CI: confidence interval
GDS: Guide Dog School of the Finnish Federation of the Visually Impaired
MLST: multilocus sequence typing
MRSA: methicillin resistant *Staphylococcus aureus*
MRSP: methicillin resistant *Staphylococcus pseudintermedius*
PFGE: pulsed-field gel electrophoresis
SCC*mec*: staphylococcal cassette chromosome *mec*
ST: sequence type
