# Supplementary figures and images for: Epidemiology of methicillin resistant Staphylococcus pseudintermedius in guide dogs in Finland
Source: Acta Vet Scand. 2015 Jul 17;57(1):37. doi: 10.1186/s13028-015-0129-8 (PMC4504442; doi:10.1186/s13028-015-0129-8)

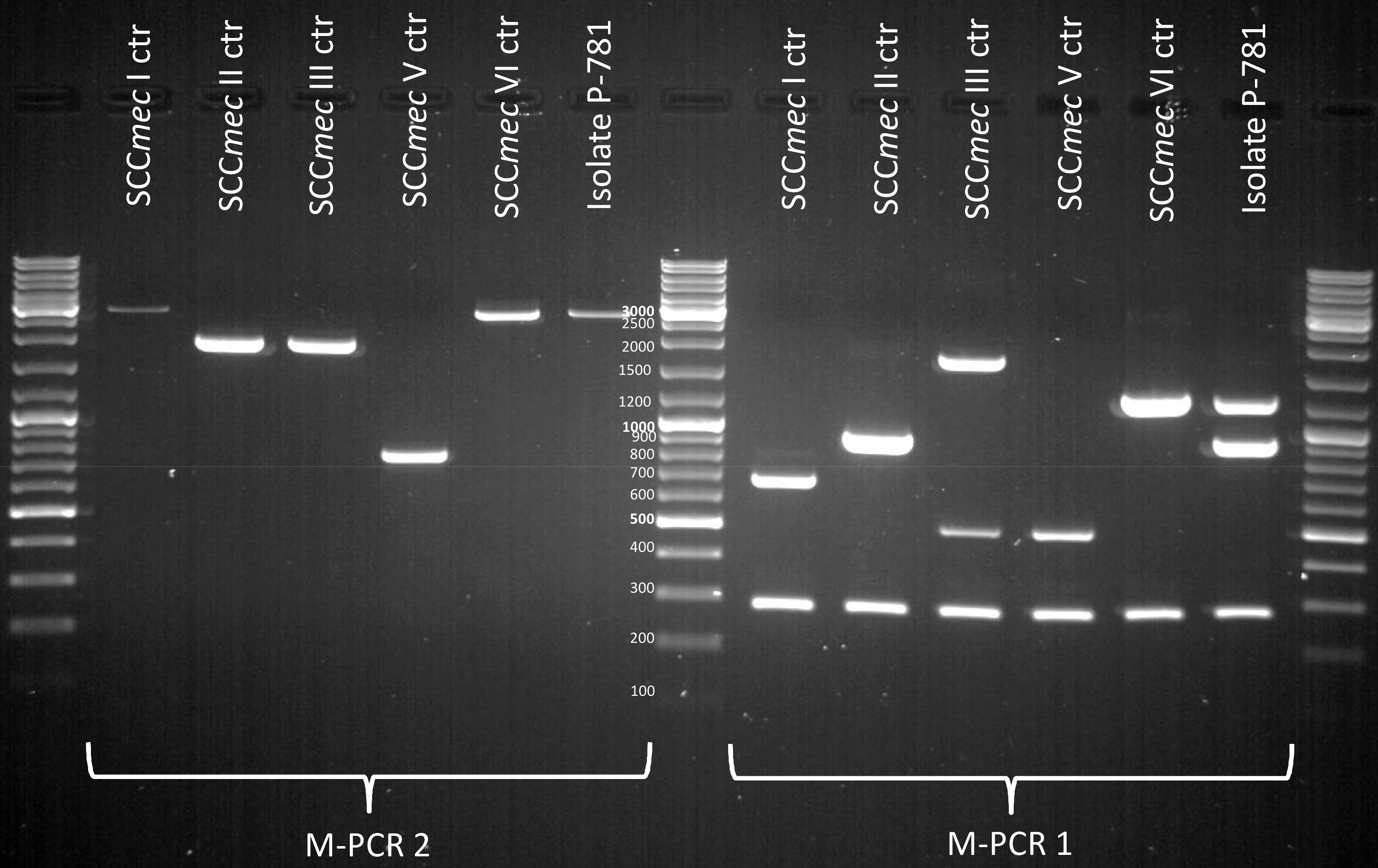

Supplement: Additional file 1: — Gel image of the products of isolate P-781 and positive controls after SCCmec PCR. Description of data: ctr = control; SCCmec I ctr: Staphylococcus aureus NCTC 10442; SCCmec II ctr: Staphylococcus aureus ATCC BAA-1720; SCCmec III ctr: Staphylococcus aureus ATCC BAA-43; SCCmec V ctr: Staphylococcus aureus JCSC 6944; SCCmec VI ctr: Staphylococcus aureus ATCC BAA-42. [file 13028_2015_129_MOESM1_ESM.png]

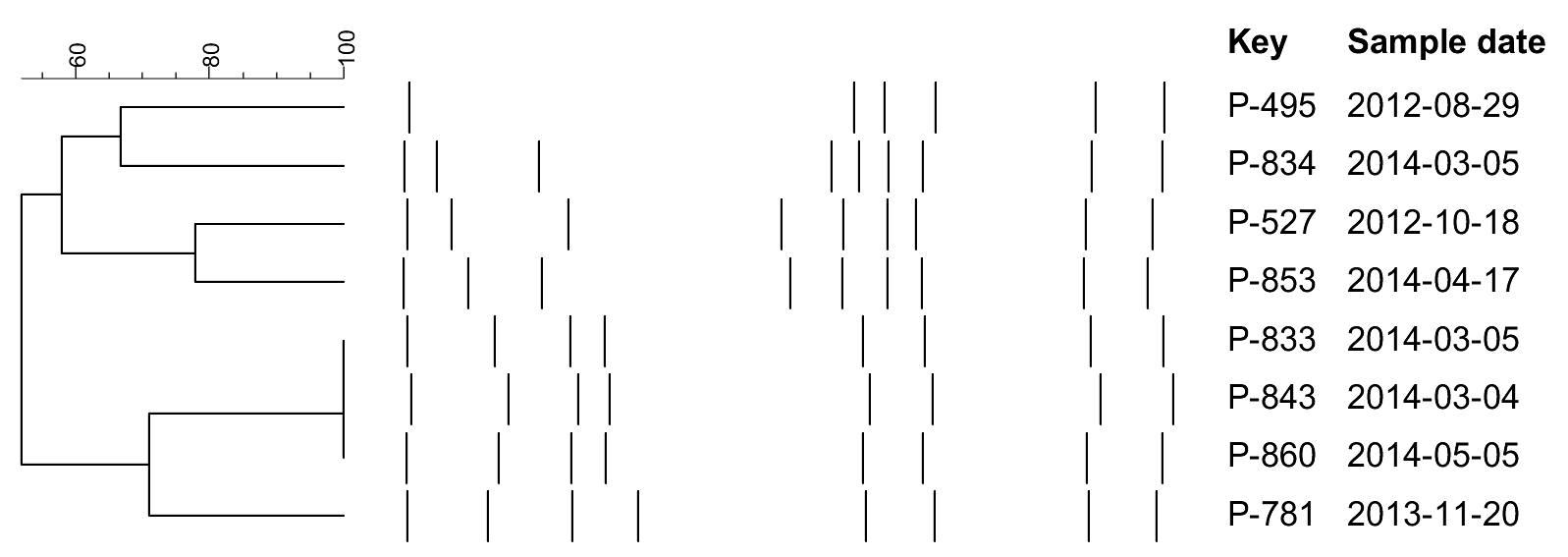

Supplement: Additional file 2: — AscI PFGE dendogram of the MRSP-isolates investigated. Description of data: All isolates were also investigated using SmaI restriction (see text). [file 13028_2015_129_MOESM2_ESM.png]
